# Association of sleep attitudes with sleep hygiene, duration, and quality: a survey exploration of the moderating effect of age, gender, race, and perceived socioeconomic status

**DOI:** 10.1080/21642850.2019.1567343

**Published:** 2019-02-11

**Authors:** Aria R. Ruggiero, Hannah D. Peach, Jane F. Gaultney

**Affiliations:** Department of Psychological Sciences, University of North Carolina at Charlotte, Charlotte, NC, USA

**Keywords:** Sleep, sleep attitudes, socioeconomic status, gender, disparities

## Abstract

**Objectives:**

Sleep health is becoming more widely accepted as a possible preventative strategy against chronic disease and negative psychosocial outcomes. It is important to understand whether attitudes towards sleep vary by demographic characteristics and how potential differences in sleep attitudes could impact sleep outcomes. The present study examined whether there were demographic differences in sleep attitudes and whether the interaction between demographic characteristics and sleep attitudes impacted sleep outcomes (e.g. sleep hygiene, duration, and quality).

**Methods:**

One hundred seventy-two adults from across the United States completed an anonymous survey on sleep and health.

**Results:**

Sleep attitudes varied according to age, gender, and race, with more positive sleep attitudes reported by older adults, women, and those who identified as White. Although positive sleep attitudes predicted more sleep and better quality sleep, this association varied as a function of several demographic characteristics. A more complex picture arose for the interaction between demographics and sleep attitudes predicting sleep outcomes.

**Conclusions:**

Future research should continue to discover for whom favorable sleep attitudes are beneficial and explore when and how sleep attitudes may be altered.

Sleep health increasingly is viewed as a crucial determinant of physical and psychosocial health and well-being (Institute of Medicine, 2006). In fact, adequate sleep has recently been highlighted for the first time as a public health priority in the Healthy People 2020 initiative aimed at improving health in the United States. While recognition of this health topic is expanding, ‘the cumulative effects of sleep loss and sleep disorders represent an under-recognized public health problem’ (Institute of Medicine, 2006, p. 1); therefore, greater attention is warranted in order to adequately address this growing public health concern. Furthermore, poor sleep that stems from lifestyle-related causes, rather than an organic disorder (i.e. sleep apnea, restless leg syndrome), affects a much larger portion of the population; yet, less research examines this construct of non-disordered inadequate sleep (e.g. Shochat, 2012), and potential reasons influencing the presence of non-disordered sleep disturbances, such as attitudes toward sleep.

## Sleep attitudes

Attitudes have been seen as modifiable predictors of a number of social and health behaviors, such as sun-protective behavior (Prentice-Dunn, McMath, & Cramer, 2009), and smoking cessation (Rise, Kovac, Kraft, & Moan, 2008). Thus, attitudes are one component that can shape health behaviors and outcomes and it is important to further study the impact that attitudes can have on other health behaviors, such as sleep hygiene and sleep outcomes. A variety of construct definitions for attitudes have been presented in the literature. According to the widely used theory of planned behavior, attitudes have been defined as the degree to which a person holds a favorable or unfavorable evaluation or appraisal of a particular behavior (Ajzen, 1991). Some studies (Knowlden, Sharma, & Bernard, 2012; Kor & Mullan, 2011) have examined attitudes as predictors of sleep and sleep hygiene within the framework of the theory of planned behavior; Knowlden et al. (2012) found that attitudes significantly predicted behavioral intentions to sleep, while Kor and Mullan (2011) reported no predictive ability of attitudes. Other literature provides a definition of dysfunctional attitudes towards sleep as ‘psychological conceptualization of insomnia,’ or ‘faulty beliefs and attitudes about sleep’ (Morin, Vallières, & Ivers, 2007, p. 1547) measured via the Dysfunctional Beliefs and Attitudes about Sleep Scale (DBAS-16; Morin et al., 2007). The DBAS has been shown to predict sleep and sleep hygiene in young adults (Yang, Chow, & Hsiao, 2011) and college students (Woodley & Smith, 2006). Yet, these definitions are limited, as sleep is both a health behavior and a biological state of consciousness that is necessary for survival, therefore a more comprehensive conceptualization is necessary for defining sleep attitudes.

Based on the ‘umbrella’ definition set forth by Eagly and Chaiken (1993, 2007); the present study defined *sleep attitudes* as the propensity to evaluate sleep with some degree of favor or disfavor that is formed, informed, and expressed by cognitive, affective, and behavioral processes (Peach & Gaultney, 2017). This conceptualization takes into account evaluative responding to an attitude object (e.g. sleep) as a tendency rather than a state or disposition, and also considers recognizes the interactive cognitive, affective, and behavioral components that capture several elements of sleep attitudes, which is different from prior definitions of sleep attitudes. For example, sleep attitudes may be shaped by beliefs about the necessity of sleep (e.g. cognitive process), positive evaluations of the benefits of sleep (e.g. affective process), and actions pertaining to sleep, such as making time for sleep in one’s schedule (e.g. behavioral process). Although sleep attitudes is a relatively novel construct with recent psychometric validation (Peach & Gaultney, 2017), it is important to continue to explore the nature of this construct considering initial evidence suggests that attitudes are predictors of sleep hygiene, duration, and quality (Peach & Gaultney, 2017; Peach, Gaultney, & Ruggiero, 2018). Given that no other studies examine the association between sleep attitudes and sleep hygiene, duration, and quality, the present study aims to build upon this nascent line of research using the present definition of sleep attitudes. Moreover, it is important to further study the predictors and outcomes of poor and inadequate sleep, especially in populations that are already disadvantaged and at risk for health problems, since poor sleep health has become a public health epidemic.

## Disparities in sleep

Sleep tends to vary across the lifespan, including recommendations for the amount of sleep one should get. Some studies have shown that aging leads to worse sleep, as measured by increased sleep latency and decreased REM sleep (Ohayon, Carskadon, Guilleinault, & Vitiello, 2004). However, results appear to be mixed. For instance, studies have also found that sleep increased in quality and duration in older adults (Grandner et al., 2012; Ohayon et al., 2004). Moreover, a recent study conducted by Fox et al. (2018) found that sleep debt (the cumulative effect of not getting enough sleep) actually decreased with age, counter to conventional wisdom that would mirror prior research that sleep gets worse as one ages (e.g. Ohayon et al., 2004). Fox et al. (2018) theorize that their findings may highlight how older adults are more likely to have more control over their sleep, in part due to fewer environmental barriers interfering with sleep (i.e. children, working less, etc.). It is possible that other factors could also be at play to support these recent findings, such as attitudes towards sleep later in life.

A number of sleep characteristics may also vary based on gender, including sleep quality, sleep duration, sleep latency, and sleep efficiency (Krishnan & Collop, 2006). Some studies have shown that women tend to have higher sleep quality, shorter sleep duration, and longer sleep latency; despite this, women tend to report overall more sleep-related complaints compared to men (e.g. Krishnan & Collop, 2006). However, other studies have found that women reported *poorer* sleep quality (e.g. Reyner & Horne, 1995), and some studies have found no gender differences in sleep at all (e.g. Voderholzer, Al-Shajlawi, Weske, Feige, & Reimann, 2003). One potential explanation for these varying results is that sleep disturbances in women are more prevalent at particular phases of the menstrual cycle (Baker & Driver, 2004), as well as other phases where hormonal fluctuations may directly impact sleep processes and regulation, such as pregnancy and menopause (e.g. Soares & Murray, 2008). However, given these inconsistent findings, it is important to further tease apart the effect that gender might have on measures of sleep, including attitudes towards sleep. It may be especially important for women to have favorable attitudes towards sleep, given their predisposition to experience altered sleep processes related to fluctuations in hormones across different phases of their cycle.

Conversely, racial and socioeconomic disparities in health and health behaviors have been well documented in recent years. For instance, a low socioeconomic status (SES) is associated with increased unhealthy behaviors such as tobacco smoking, physical inactivity, and poor nutrition (e.g. Pampel, Krueger, & Denney, 2010). Identifying as a racial minority has been linked to earlier onset of illness and more severe illnesses in comparison to Whites (e.g. Williams, Mohammed, Leavell, & Collins, 2010). Importantly, evidence also suggests that racial disparities in health exist at every level of SES (Williams et al., 2010). Thus, it is crucial to continue exploring important contributors of health, how they vary based on race and SES, and the implications of any differences observed in health behaviors.

Despite the relevance of racial and SES disparities in health behaviors, few studies have examined their combined association with sleep (Durrence & Lichstein, 2006). Research suggests that African Americans take longer to fall asleep, report poorer sleep quality, have more light and less deep sleep, and nap more often and longer (Durrence & Lichstein, 2006; Petrov & Lichstein, 2016). African Americans also have 37% greater odds of reporting insufficient sleep compared to Whites (Williams et al., 2016), and have shorter sleep duration and lower sleep efficiency (Mezick et al., 2008). Additionally, African Americans may engage in behaviors or experience environmental conditions that do not promote effective, sufficient sleep, such as more frequent naps, consuming an excess of rich, fatty meals, and living in environments that tend to be noisier and have less temperature control (Durrence & Lichstein, 2006). The combination of poor sleep and engaging in other generally unhealthy behaviors could lead to a compounding effect of negative health outcomes within this ethnic group. Additionally, in their review of ethnic differences in sleep, Durrence and Lichstein (2006) highlighted a need to specifically focus on ethnicity, age, and gender in future sleep research, which has informed the present research. These sleep behaviors/outcomes may also vary by other demographic identities that individuals hold, such as age, gender, and perceived SES. Moreover, most of this limited body of research has only compared African Americans with Whites, and research involving other ethnic minority groups is sparse. Whinnery, Jackson, Rattanaumpawan, and Grandner (2014) found that Hispanic/Latinos in their sample were 3.5 times as likely to be considered a very short sleeper (<5 h), while those who identified as Asian were 5 times as likely. Additionally, Fox et al. (2018) found that participants who identified as Latino/a were more likely to have sleep debt than other races/ethnicities.

It is important to keep in mind that race tends to be conflated with SES (Jackson & Williams, 2006) so teasing the two apart during social science research can be a challenge. It has been found that differences in SES across racial groups are a key contributor to racial disparities in health outcomes (Williams et al., 2010). Moreover, research has found that some of the racial disparities in health are associated with differences in SES, but even after factoring in SES, racial disparities in health practices and health outcomes often remain (Franks, Meldrum, & Fiscella, 2006). This is a key consideration given that some disparities in sleep have been studied as a function of SES. For instance, low SES has been linked with higher rates of overall sleep disturbance, such as difficulty falling or staying asleep, and getting less sleep at night (Grandner et al., 2013). Studies have also demonstrated that low SES is linked to insomnia (Gellis et al., 2005), and sleep quality (Hall, Bromberger, & Matthews, 1999), and that sleep quality has been shown to mediate the relationship between income and physical and mental health (Moore, Adler, Williams, & Jackson, 2002). Despite these findings, the degree to which race and SES uniquely contribute to facets of sleep remains uncertain (Mezick et al., 2008). Additionally, most of the research examining racial/ethnic disparities in sleep only factor in certain common facets of sleep, such as sleep duration and sleep quality, and not attitudes surrounding sleep practices and outcomes. Thus, it will be important to also examine how demographic characteristics are associated with sleep attitudes, since this is a novel line of research.

## The present study

If attitudes are historically modifiable for a number of health behaviors, it is possible that sleep attitudes could have implications for shaping sleep hygiene practices and sleep outcomes. However, it is not known whether sleep attitudes are consistent across demographic groups, or whether interactions of attitudes with demographic characteristics predict sleep behavior or outcomes. The purpose of the present study was to examine age, gender, race, and SES differences in sleep attitudes, and explore whether these demographic variables moderate any differences in sleep attitudes as predictors of sleep hygiene, sleep duration, and sleep quality. Any disparities observed could help inform future behavioral interventions targeting sleep attitudes in order to improve sleep outcomes for certain groups. This is important since sleep health is linked with a number of different health outcomes, including obesity, heart disease, and all-cause mortality. Improving sleep outcomes for certain groups could also have trickle-down effects and have the potential to positively affect other health outcomes in these groups. The primary purpose of the study was exploratory rather than hypothesis testing given the limited body of research on sleep attitudes relating to demographic characteristics, and sleep practices and outcomes. However, based on previous findings (e.g. Durrence & Lichstein, 2006; Fox et al., 2018; Grandner et al., 2013; Krishnan & Collop, 2006), we expected to find that demographic factors differentially predicted sleep attitudes, and would interact with sleep attitudes in predicting sleep outcomes.

## Method

### Participants

Participants (*N* = 172) consisted of adults who lived in the United States. The sample consisted of participants self-identifying race/ethnicity, which included 119 Whites, 22 African-Americans, 20 Asians, 7 Hispanic/Latino/as, 1 Native American, and 4 who identified as mixed race. Table 1 contains additional demographic and descriptive data for this sample. Participants were recruited through Amazon’s Mechanical Turk (MTurk) system and gave consent prior to data collection. A power analysis indicated a minimum sample size of 68 to find a moderate effect size with the power of .80. Inclusionary criteria specified that in order to participate in the present study, participants must be at least 21 years of age and reside in the United States.

**Table 1.** Bivariate correlations between predictors and outcome variables.

	%(*N*)	*M*(SD)	1	2	3	4	5	6	7	8	9
1. Age		33.31(9.86)	–								
2. Gender^a^ (% female)	41(70)		.18*	–							
3. Race^b^ (self-identified minority)	32(54)		.19*	–.02	–						
4. SES		4.76(1.76)	.07	–.12	.09	–					
5. Sleep attitude		5.14(.80)	.24**	.21**	.18*	.02	–				
6. Sleep hygiene		78.21(24.41)	–.10	–.07	–.22**	–.40	–.58**	–			
7. Sleep duration – weeknights		.64 (.71)	.11	.06	–.21**	–.11	–.27**	.30**	–		
8. Sleep duration – weekends		.47(.73)	.09	.16*	–.12	–.13	–.30**	.21**	.77**	–	
9. Sleep quality – weeknights		5.65(3.85)	–.01	.10	–.16*	–.05	–.33**	.60**	.63**	.48**	–
10. Sleep quality – weekends		5.45(3.77)	–.01	.12	–.15*	–.06	–.36**	.59**	.59**	.57**	.96**

Notes: Higher scores indicate *shorter* sleep duration; Higher scores indicate *poorer* sleep quality. A score of 0 for sleep duration indicates >7 hours of sleep; 1 = 6–7 hours; 2 = 5–6 hours; 3 = <5 hours. A score of 5 or greater for sleep quality indicates a ‘poor’ sleeper. Correlations between dichotomous (race, gender) and continuous variables are point-biserial

*p < .05.

**p < .01.

^a^Males = 0, Females = 1.

^b^Self-identified minority status = 0, Self-identified white = 1.

### Materials

Participants reported age, gender, race, perceived SES, use of any medication that affected sleep or sleepiness, and if the participant had ever been diagnosed with a sleep disorder. Data on age, gender, race, and SES were used specifically in the current study. SES was measured using the MacArthur Scale of Subjective Social Status (Adler, Epel, Castellazzo, & Ickovics, 2000), which captures the common sense of one’s perceived social status across SES indicators (i.e. income, occupation, education, etc). Participants were presented with a ‘social ladder’ where they were asked to mark where they felt they were on that ladder relative to other people in the United States at this time in their lives, ranging from 1 to 10 (lowest to highest). Participants in the present study endorsed values ranging from 1 to 9. Perceived SES was chosen for this study because it captures the way individuals believe they compare to others in regards to SES, regardless of their objective SES levels (Dennison, 2016). Additionally, prior research has suggested that perceived SES is a better predictor of health outcomes even after adjustment for objective SES measures (e.g. Adler & Snibbe, 2003).

#### Attitudes

Sleep attitudes were measured using the Charlotte Attitudes Towards Sleep Scale (CATS; Peach & Gaultney, 2017). This 10-item measure includes two dimensions of sleep attitudes (sleep benefits/enjoyment and sleep as a time commitment). Examples of items representing sleep benefits/enjoyment and sleep as a time commitment, respectively, includes, ‘Getting a good night’s sleep makes me happy’ and, ‘I sleep less so I have more hours during the day to get work accomplished’. Responses range from 1 (strongly disagree) to 7 (strongly agree). Items that are negatively worded (i.e. indicating more unfavorable attitudes towards sleep) were reverse coded. Items were averaged, so that higher scores indicate more favorable attitudes towards sleep. In the present study, internal consistency estimated for the total scale (*α* = .79) was reasonable. This estimate mirrors the scale’s internal consistency (*α* = .76) found during scale validation. The present sample had a mean sleep attitude of 5.14 (SD = .80, Range = 3–7).

#### Sleep hygiene

Sleep hygiene behaviors were measured using the Sleep Hygiene Practice Scale (SHPS; Lin, Cheng, Yang, & Hsu, 2007). The SHPS consists of 30 items measuring four hygiene domains, including arousal-related behaviors, sleep scheduling and timing, eating/drinking behaviors, and sleep environment. Participants rate how frequently they engage in each practice. Responses range from 1 (never) to 6 (always). Items were summed across domains to yield a total score, and four subscores were created by summing the items from the individual subscales. Higher scores indicate worse sleep hygiene. Internal consistency for the present study for the total scale was good (α=.93). The present sample had a mean hygiene score of 78.21 (SD = 24.41, Range = 30-140).

#### Sleep outcomes

Sleep outcomes in this study consisted of subjective sleep quality and quantity and were measured via The Pittsburgh Sleep Quality Index (PSQI; Buysse, Reynolds, Monk, Berman, & Kupfer, 1989). The PSQI is a widely accepted, reliable, and validated standardized measure of sleep quality. The scale includes 19 self-rated questions where participants are asked about their sleep over the past month. In the present study, self-reported sleep quality was measured by the global score, in which higher scores indicated worse sleep quality (ranges from 0–16). Self-reported sleep duration was measured via the PSQI component score for sleep duration based on the item ‘How many hours of actual sleep do you get at night? (This may be different than the number of hours you spend in bed).’ Higher scores indicated shorter sleep durations based on how the component score is scored for the measure (>7 h = 0; 6–7 h = 1; 5–6 h = 2; <5 h = 3). Inconsistent weeknights vs. weekend sleep patterns have been frequently reported among adolescents (e.g. Wolfson & Carskadon, 1998); research has also found such discrepancies among adults (e.g. Gaultney, 2014). As a result, the present study chose to examine sleep quantity and sleep quality separately by weeknights and weekend nights to account for this potential discrepancy in sleep patterns.

### Procedure

Participants completed a series of online questionnaires, including questions on demographic information, alcohol use, depression, stress, sleep attitudes and outcomes, and health behaviors. Participants were first shown a screen with a consent statement, acknowledging that they were at least 21 years of age and living in the United States, and they indicated their consent by continuing on to the survey questions. The institutional review board of the university at which this study was conducted approved this study (IRB #17-0226).

### Design and plan of analysis

Since the purpose of the project was exploratory, no hypotheses were tested. Survey questions asked participants to self-report demographic information along with describing their sleep and responding to the sleep attitudes measure.

Participants’ race was collected via an open-ended prompt and was manually coded by two different researchers to ensure accuracy and inter-rater reliability in coding. The variable for race included in all analyses was dummy coded, and dichotomized White as a reference group (set as 1 when dummy coded) and all other participants as an ethnic Minority group. Thus, the Minority variable (set as 0 when dummy coded) consisted of anyone who identified as African-American, Asian, Latino/a, Native American, or mixed race. Additionally, participants who identified as having another gender aside from male or female (*N* = 1) were excluded from the analyses given the small sample size and inability to perform accurate group comparisons with only one member of a different gender identity, resulting in a total of 172 participants included in the analyses involving gender. Therefore, identifying as male was represented as 0 and identifying as female was represented as 1 in the analyses.

When preparing the data for all moderation analyses, all independent variables were mean centered and entered into the first step of the regression analysis. Two-way interaction terms were calculated by multiplying the centered variables, then entered into the second step of the regression analysis. The same procedure was performed for three-way interaction terms, which were entered into the third step of the regression analyses they were tested for.

Given both the exploratory nature of this study and the intersecting nature of the role of demographics on health behaviors and sleep in the literature, the authors felt it was plausible to explore not only two-way interactions but also three-way interactions. It was of particular interest to examine the interactions including sleep attitudes since that was the primary focus of the paper. Moreover, this decision was made in order to know what relationships could be ruled out for future research.

Additionally, sleep duration and quality were divided into weeknight and weekend night sleep. A PSQI global score (measuring sleep quality) was generated for weeknights and weekends separately, and a PSQI component score (measuring sleep duration) was also generated for weeknights and weekends separately. Weeknights and weekends were considered separately because many studies of adolescents (e.g. Hasler et al., 2012) and some of adults (e.g. Gaultney, 2014) indicate there can be notable time-of-week discrepancies in sleep. In addition, paired samples *t*-tests comparing weeknight vs. weekend sleep outcomes indicated a significant difference in weeknight sleep duration (*M* = .64, SD = .71) and weekend sleep duration (*M* = .48, SD = .73) for this study; *t*(172) = 4.22, *p* < .01. Moreover, there was a significant difference in weeknight sleep quality (*M* = 5.65, SD = 3.85) and weekend sleep duration (*M* = 5.46, SD = 3.77); *t*(172) = 2.48, *p* < .05.

Multiple regression analyses first examined whether there were demographic differences in sleep attitudes. Next, sleep hygiene was regressed onto sleep attitudes, in addition to examining whether sleep attitudes interacted with the demographic variables to predict sleep hygiene. Finally, we regressed sleep outcomes (duration and quality) onto sleep attitudes and the demographic interaction terms. All analyses utilized multiple regression models and report unstandardized coefficients. Lastly, all additional interactions (non-significant) can be found in the Appendix.

### Ethics statement

This study was reviewed by the university's Institutional Review Board (IRB). All participants were shown a consent statement prior to data protections, and indicated willingness to continue with the anonymous, online survey by clicking “I agree” and continuing to the survey questions. Because no identifying information about participants was recorded, the IRB identified the study to be Exempt from IRB oversight under 45 CFR 46.101(b).

## Results

Table 1 indicates descriptive data and presents bivariate correlations between predictor and outcome variables used to initially inspect the data. Correlations were in the expected direction (e.g. better sleep attitudes were associated with longer sleep duration, better sleep quality, better sleep hygiene; longer sleep duration was associated with better sleep quality; better sleep hygiene was associated with longer sleep duration and better sleep quality; and identifying as a Minority was associated with shorter sleep duration and poorer sleep quality).

### Sleep attitudes

**Figure 1.**
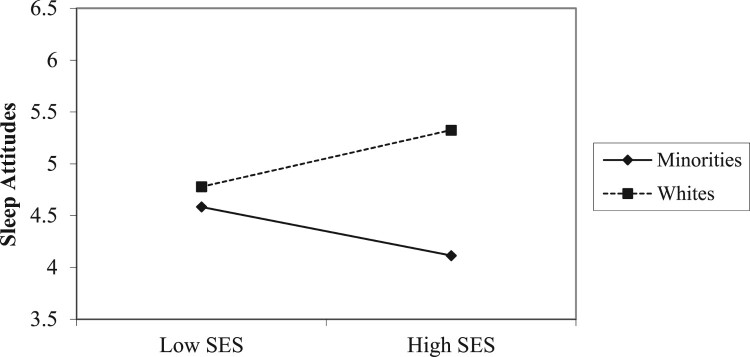
SES x race predicting sleep attitudes.

First, to examine whether there were any relationships between age, gender, race, or SES and sleep attitudes, we ran multiple regression analyses looking at the main and interaction effects of the demographic variables on sleep attitudes (see Table 2). Main effects of gender and age on sleep attitudes indicated that females and older adults had more positive attitudes towards sleep. Race trended towards significance, such that Whites had more positive attitudes than Minorities. Additionally, a significant interaction (Figure 1) of race X SES on sleep attitudes indicated that among individuals of a lower SES, race differences in attitudes were small. However, Minority individuals who reported higher SES had the most negative sleep attitudes, while White individuals of a higher SES had the most positive sleep attitudes.

**Table 2.** Main effects and interactions on sleep attitudes.

Model	Outcome variable	*b*	S.E.	*R*^2^	Δ*R*^2^
Step 1				0.10^b^	0.10^b^
	(Intercept)	4.82	0.14		
	Age	0.02^a^	0.01		
	Gender	0.32^a^	0.15		
	Race	0.28^c^	0.15		
	SES	0.01	0.04		
Step 2				0.16^b^	0.05
	(Intercept)	4.78	0.18		
	Age	0.02	0.02		
	Gender	0.31	0.26		
	Race	0.32	0.20		
	SES	–0.14^c^	0.08		
	Age X Gender	–0.002	0.02		
	Age X Race	0.004	0.02		
	Age X SES	0.000	0.004		
	Gender X Race	–0.02	0.31		
	Gender X SES	–0.04	0.09		
	Race X SES	0.25^b^	0.09		

Notes: *N* = 172. *b* = unstandardized regression weight; Δ*R*^2 ^= Change in *R*^2^ from prior model.

^a^*p* < .05.

^b^*p* < .01.

^c^*p* = .06.

**Table 3.** Main effects and interactions on sleep hygiene.

Model	Outcome variable	*b*	S.E.	*R*^2^	Δ*R*^2^
Step 1				.36^b^	.36^b^
	(Intercept)	82.17	3.16		
	Age	0.12	0.17		
	Gender	1.84	3.24		
	SES	–0.18	0.88		
	Race	–6.70^a^	3.38		
	Sleep Attitudes	–15.00^b^	1.71		
Step 2				.39^b^	.03^a^
	(Intercept)	80.53	3.19		
	Age	0.08	0.16		
	Gender	1.07	3.20		
	SES	–0.63	0.91		
	Race	–4.92	3.43		
	Sleep Attitudes	–18.80^b^	3.17		
	Gender X Sleep Attitudes	7.03^b^	3.35		
	SES X Sleep Attitudes	–1.61^c^	0.88		
	Race X Sleep Attitudes	1.88	3.52		

Notes: *N* = 172. *b* = unstandardized regression weight; Δ*R*^2 ^= Change in *R*^2^ from prior model.

^a^*p* < .05.

^b^*p* < .01.

^c^*p* = .06.

**Table 4.** Sleep duration.

Model		Outcome variable	*b*	S.E.	*R*^2^	Δ*R*^2^
*Weeknights*						
Step 1					0.12^b^	0.12^b^
		(Intercept)	0.74	0.10		
		Age	.15^b^	0.01		
		Gender	0.15	0.11		
		SES	–0.03	0.03		
		Race	–0.23^a^	0.11		
		Sleep Attitudes	–0.20^a^	0.06		
Step 2					0.14^b^	0.02
		(Intercept)	0.66	0.13		
		Age	–.01	0.02		
		Gender	0.36^c^	0.20		
		SES	0.02	0.06		
		Race	–0.13	0.16		
		Sleep Attitudes	–0.23^c^	0.12		
		Gender X Race	–0.30	0.24		
		Gender X SES	–0.25	0.07		
		Gender X Sleep Attitudes	–0.02	0.12		
		SES X Race	–0.06	0.07		
		SES X Sleep Attitudes	0.04	0.03		
		Race X Sleep Attitudes	0.06	0.13		
		Age X Sleep Attitudes	–.01	.01		
		Gender X Age	–.01	.01		
		Age X Race	.03	.02		
		Age X SES	–.01^b^	.01		
*Weekends*						
Step 1					0.15^b^	0.15^b^
		(Intercept)	0.46	0.11		
		Age	.01^b^	.01		
		Race	–0.14	0.11		
		SES	–0.04	0.03		
		Gender	0.29^b^	0.11		
		Sleep Attitudes	–0.25^b^	0.06		
Step 2					0.20^b^	0.05
		(Intercept)	0.30	0.13		
		Age	0.08^b^	0.16		
		Race	0.05	0.16		
		SES	–0.04	0.06		
		Gender	0.57^b^	0.20		
		Sleep Attitudes	–0.48^b^	0.12		
		Gender X Race	–0.41	0.24		
		Gender X SES	–0.003	0.07		
		Gender X Sleep Attitudes	0.02	0.12		
		SES X Race	–0.01	0.07		
		SES X Sleep Attitudes	0.03	0.03		
		Race X Sleep Attitudes	0.33^a^	0.13		
		Age X Sleep Attitudes	–.01	.01		
		Gender X Age	–.01	.01		
		Age X Race	.03	.02		
		Age X SES	–.01^b^	.01		

Notes: *N* = 172. *b* = unstandardized regression weight; Δ*R*^2 ^= Change in *R*^2^ from prior model.

**^a^***p* < .05.

^b^*p* < .01.

^c^*p* = .06.

### Sleep hygiene

**Figure 2.**
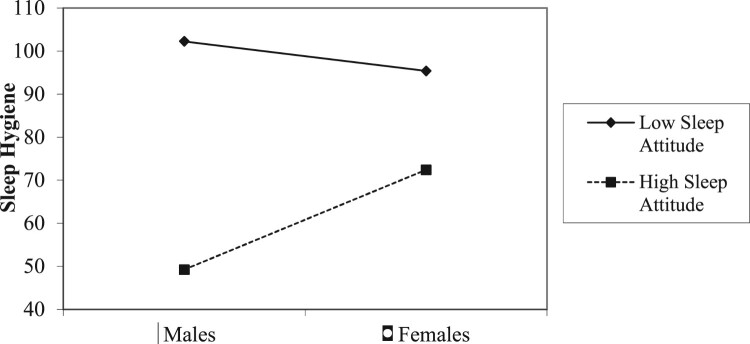
Gender x sleep attitudes predicting sleep hygiene. Note: Higher scores indicate *worse* sleep hygiene.

Next, we looked at whether sleep attitudes were associated with sleep hygiene (sleep-promoting behaviors), and whether sleep attitudes interacted with age, gender, race, or SES to predict sleep hygiene. Main effects found for race and sleep attitudes on sleep hygiene indicated that Whites and participants with more positive sleep attitudes reported better sleep hygiene. A significant interaction of gender X sleep attitudes on sleep hygiene (Figure 2), indicating that males with more negative sleep attitudes had worse sleep hygiene than those with more positive sleep attitudes. Sleep attitudes appeared to be less associated with sleep hygiene among women (Table 3).

### Sleep duration

We next examined whether sleep attitudes predicted sleep outcomes, including duration and quality, collected separately for weeknight and weekend, and whether there were interactions of sleep attitudes with the demographic variables in predicting sleep outcomes (Table 4).

### Weeknight sleep duration

Significant main effects of age, race, and sleep attitudes on weeknight duration, in which older adults slept longer, Minorities had shorter sleep duration during the week than Whites, and more positive sleep attitudes were linked to longer sleep duration. No interaction effects reached significance.

### Weekend sleep duration

Significant main effects of age, gender, and sleep attitudes were also found on weekend duration. Positive sleep attitudes again associated with longer sleep. In addition, women reported longer sleep on weekends as well as older adults.

**Figure 3.**
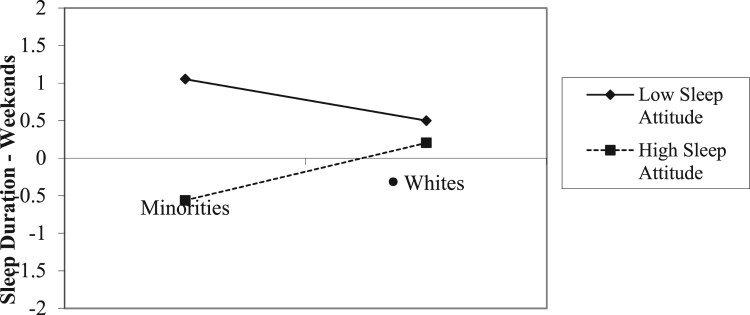
Race x sleep attitudes on sleep duration – weekends. Note: Higher scores indicate *shorter* sleep duration.

**Figure 4.**
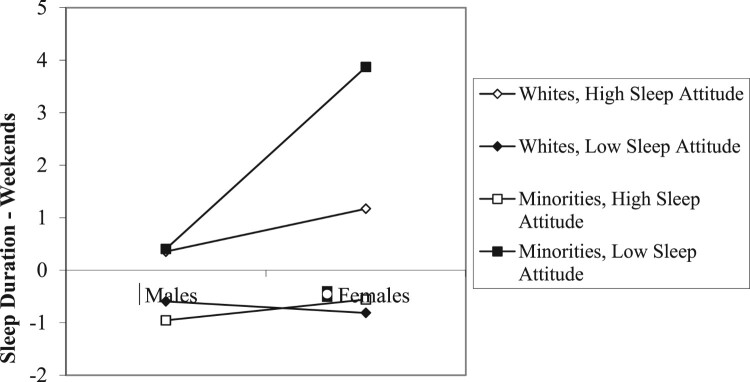
Gender x race x sleep attitudes on sleep duration – weekends. Note: Higher scores indicate *shorter* sleep duration.

A significant race X sleep attitudes interaction (Figure 3) was modified by a significant gender X race X sleep attitudes interaction (Figure 4). The race X sleep attitude interaction on weekend duration appeared to be most pronounced among women. Minority women with lower sleep attitudes reported less weekend sleep than did those with a positive sleep attitudes. While sleep attitudes appeared to play a similar role among White females, the difference in duration as a function of sleep attitudes was not as great.

**Figure 5.**
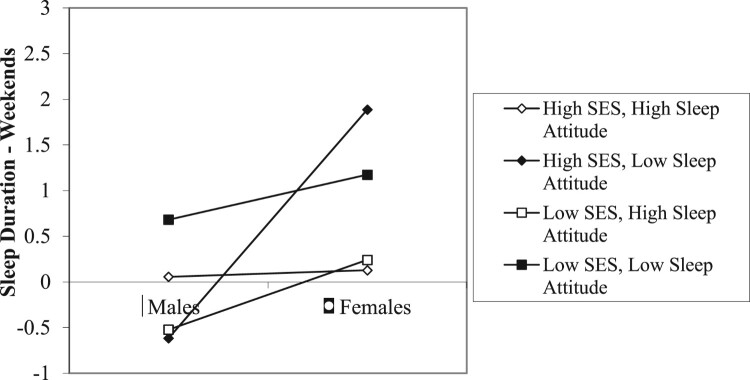
Gender x SES x sleep attitude on sleep duration – weekends. Note: Higher scores indicate *shorter* sleep duration.

Additionally, a gender X SES X sleep attitudes interaction (Figure 5) also reached significance. This significant interaction indicated that sleep attitudes predicted weekend duration most strongly among participants reporting higher SES. Sleep attitudes as a predictor of weekend duration was opposite among higher SES males and females. Among males, less positive sleep attitudes predicted less sleep among individuals who reported a lower SES. Among females with less positive sleep attitudes, those with a *higher* SES reported getting the *least* sleep of all.

### Sleep quality

Analysis of weekend and weeknight sleep quality (analyzed separately) produced significant main effects of sleep attitudes (Weeknight: *b* = −1.44, *p *< .05, *R*^2 ^= .15; Weekend: *b* = −1.52, *p *< .05; R^2^=.17) and gender (Weeknight: *b *= 1.31, *p *< .05, *R*^2^ = .15; Weekend: *b *= 1.45, *p *< .05, *R*^2^ = .17). More positive attitudes predicted better sleep quality, and women reported poorer sleep quality both on weeknights and weekends. No significant interactions were found.

## Discussion

The present study explored whether there were demographic differences in sleep attitudes, and whether these variables moderated sleep attitudes as a predictor of sleep outcomes. Overall, more positive sleep attitudes were associated with longer sleep duration, better sleep quality, and better sleep hygiene. However, several interactions modified these main effects.

### Demographic predictors of sleep attitudes

Older adults, women, and White participants reported more favorable sleep attitudes. However, the race difference depended on SES, in that race made little difference in mean sleep attitude among those with lower SES, but was pronounced among those with higher SES. To some extent, these findings are consistent with those of other health-related behaviors. Previous research has found that older adults and women generally have more positive health-related behaviors, such as engaging in screening health checks (Deeks, Lombard, Michelmore, & Teede, 2009). Further, research has shown that health-related behaviors, including sleep, tend to ‘cluster,’ particularly for women and older adults compared to men (Liu et al., 2016). Given an association of health behaviors with health attitudes (Ajzen & Timko, 2010), it is plausible that women and older adults would also exhibit more positive sleep attitudes. Older adults may be more health conscious than younger adults in order to prevent disease and increase lifespan. Women tend to manage their family’s health, especially which could help to explain their more positive sleep attitudes (Deeks et al., 2009).

The present study also found that White participants had more favorable sleep attitudes than Minority participants. Previous research supports observed race/ethnicity difference in health attitudes (Conner, Koeske, & Brown, 2009), and demonstrated that Whites are more likely to believe that they are impaired by health problems than Minority individuals (Shive et al., 2006). These racial differences in sleep attitudes may reflect differences in SES, medical conditions, and discrimination, which have been proposed to explain differences in sleep outcomes (Petrov & Lichstein, 2016). The present study also indicated that SES modified race differences in sleep attitudes. Those with lower perceived SES may have limited perceived control, regardless of race, about one’s ability to receive enough good quality sleep by controlling one’s sleeping and living environment. Those of lower SES often live in neighborhoods that have more light, noise, and generally disturbing and unsafe conditions, with reduced ability to control such conditions. Thus, sleep attitudes may be less salient in an environment that one cannot control. If that is the case, perceived environmental control may elevate the value of sleep.

### Sleep attitudes as a predictor of sleep hygiene

The present indicated that more positive sleep attitudes were associated with better sleep hygiene behaviors. This relationship was modified by gender, such that sleep attitudes appear to better predict sleep hygiene behaviors among men. Prior research in children and adolescents have mixed results regarding gender differences in sleep hygiene, with some studies noting that girls have poorer sleep hygiene (Galland et al., 2017), and others that boys have poorer sleep hygiene (Sadeh, Raviv, & Gruber, 2000). To our knowledge, gender differences in sleep hygiene have not been reported among adults. If, at least during childhood and adolescence, males have poorer sleep hygiene than females, it would be plausible that gender differences in sleep attitudes may have a larger downstream association with sleep via differences in hygiene. This is speculative, however, given that research on gender differences in sleep hygiene in adults has not been reported.

Perceived gender roles may also be a factor. The influence of male gender roles on sleep attitudes may reflect pressure to uphold masculinity. Studies suggest that men may have different views of the meaning and values of sleep shaped by popular culture that equates being male with a lack of regard for sleep, or a mantra that ‘sleep is for wimps who can’t take the pace’ (Meadows, Arber, Venn, & Hislop, 2008, p. 697). For women, who presumably do not have to overcome a social norm for viewing sleep positively, sleep attitudes may be less salient to engaging in behaviors dedicated to improving sleep. Future research should focus on examining gender differences in sleep hygiene in adults as a potential mediator in order to increase understanding of sleep attitudes as a predictor of sleep outcomes.

### Sleep attitudes as a predictor of sleep duration and quality

Positive sleep attitudes significantly predicted greater sleep duration on both weeknights and weekends. Results also indicated significant interactions of gender X race X sleep attitudes and gender X SES X sleep attitudes for weekend sleep duration. As illustrated in Figure 3, sleep attitudes did not appear to make a large difference in weekend sleep duration among White participants, but was more evident among Minority participants. However, when the role of sleep attitudes was examined by race/ethnicity and gender (Figure 4), sleep attitudes were a strong predictor of weekend duration among Minority women, such that individuals with less positive sleep attitudes slept the *least* on weekends among the four groups examined. Previous research has highlighted, for instance, that individuals identifying as African-American, Latino/a, or Asian have shorter sleep duration in general than individuals identifying as White (e.g. Whinnery et al., 2014). Research regarding gender differences in sleep outcomes have produced inconsistent findings, with some demonstrating that women tend to have shorter sleep duration than men (e.g. Krishnan & Collop, 2006), while others have found no gender differences in sleep outcomes (e.g. Voderholzer et al., 2003). Perhaps gender differences in sleep depend more on one’s race/ethnicity and this intersectionality may compound the role of sleep attitudes on sleep duration.

Given that female Minority participants with less positive sleep attitudes reported the least amount of sleep on weekends, it is possible that these individuals did not feel the need to ‘catch up’ on sleep on weekends, since sleep may not be as much of a priority. Alternatively, research has highlighted that older African American women had the highest prevalence rates of insomnia compared to White women (Foley, Monjan, Izmirlian, Hays, & Blazer, 1999) and that African Americans engage in poorer sleep hygiene behaviors than Whites (e.g. Durrence & Lichstein, 2006), which would interfere with sleep duration. Another possibility is demographic differences in home responsibilities, which could interfere with getting more sleep on weekends as a means to ‘catch up’ from the week prior. For example, identifying as not only a mother, but also a single mother, may have an impact on how much sleep one is able to get. According to the 2010 Population Reference Bureau, 27% of Latina children and 52% of African American children live in single-mother families. There are a number of possible reasons for this finding, and additional research is needed to determine reasons *why* individuals of certain intersecting identities may not be getting enough sleep.

The gender X SES X sleep attitudes interaction (Figure 5) indicated that women with more positive sleep attitudes received more weekend sleep than those with less positive sleep attitudes. The most dramatic gender difference was among those with high SES and less favorable sleep attitudes. High SES males with less favorable sleep attitudes reported more weekend sleep, whereas women with high SES and less favorable sleep attitudes received the least amount of sleep on weekends. The role of high SES in predicting sleep attitudes and sleep outcomes was not always beneficial for some individuals, as might be initially suspected.

Although higher SES is generally associated with better health behaviors and outcomes (Pampel et al., 2010), the present data indicated that, particularly for women and Minorities, perceiving a higher SES *negatively* predicted weekend sleep duration (Figure 5) and sleep attitudes (Figure 1), respectively. One possibility is that a higher SES could allow individuals to afford more electronic devices and better Internet access. Perhaps women of high SES who placed lower value on sleep (less positive sleep attitudes) engaged in more screen time during weekends *instead* of getting more sleep. For instance, a study by Jago et al. (2014) highlighted that there were weekday-weekend discrepancies in the amount of screen time that parents engaged in. It is also possible that women in general engaged in more screen time than men. Additionally, a higher perceived SES could be associated with higher levels of stress due to a demanding job and little work-life balance. Prior research has shown that stress is considered to be the main cause of primary insomnia (Morin, Rodrigue, & Ivers, 2003). Thus, it is plausible that the association between a higher perceived SES and shorter weekend sleep duration could be influenced by stress relating to *maintaining* a higher SES.

Lastly, more positive sleep attitudes predicted better sleep quality on both weeknights and weekends. This finding is consistent with previous research on the criterion validity of the sleep attitudes construct, which demonstrated a correlation of -.34 between sleep quality and sleep attitudes (Peach & Gaultney, 2017). Race, gender, or SES did not modify the main effect of sleep attitudes on sleep quality. Given that the present study also found that more favorable sleep attitudes predicted better sleep hygiene, it is possible that sleep hygiene may serve as an indirect path in the relationship between sleep attitudes and sleep quality. Future research should test this model to clarify the relationship between sleep attitudes and sleep outcomes.

### Strengths and limitations

The authors recognize that the nature of the data means that any conclusions drawn here are tentative and require further support. A potential limitation is demographic variables could reflect group differences that have nothing to do with gender, race or SES, and more to do with differences in the freedom to make choices about when and how much one sleeps. The present study did not take into account sleep attitudes of other members of the household. For example, a partner who does not value sleep may not take steps to facilitate either their own sleep or that of the participant. Therefore, the differences reported may reflect factors not yet considered.

Interpreting the findings regarding weekend sleep is not clear. Weekend sleep may be beneficial (raising overall weekly duration; e.g. Im et al., 2017) or maladaptive (indicating greater weekend sleepiness due to greater inconsistency in bedtime; e.g. Sun, Ling, Lee, & Li, 2017). Evaluation of more weekend sleep as ‘better’ or ‘worse’ may also depend on how better or worse is operationalized. Given the findings that more positive attitude was associated with more sleep during the week and better sleep quality, we have continued this theme, assuming that ‘more sleep any time of the week is better,’ but other interpretations are possible.

Additionally, the way in which race was dummy coded into two groups, consisting of White and ethnic Minority, can be considered a limitation, as well. Given the nascent nature of this study, categorizing the racial/ethnic groups in this manner was a preliminary way to begin to understand how sleep attitudes vary based on race/ethnicity. Future research should study disparities in sleep attitudes further by comparing each ethnic group separately, and ensuring a larger sample is collected for each ethnic minority group in order to study more detailed comparisons between ethnic groups. Similarly, the relevance of sleep attitudes among individuals who classify their gender as non-binary will also need to be explored.

Previous work involving sleep attitudes has focused only on college students (Peach & Gaultney, 2017). The present study broadened the applicability of sleep attitudes to a sample that has greater generalizability to the United States population, thereby expanding the study of sleep attitudes among a wider age range. However, according to Paolacci and Chandler (2014), participants recruited via MTurk tend to be younger (*M* = 30 years), overeducated, underemployed, less religious, and more liberal than the general population. Additionally, the researchers found that although the participant pool tends to be more diverse than the average college student population, Asians tend to be overrepresented, and Blacks and Hispanics are underrepresented relative to the population as a whole. So although the present sample from MTurk was likely more representative than a sample of college students, it is uncertain whether it was generalizable to the United States adult population as a whole. These data extended previous work (Peach & Gaultney, 2017) by examining whether demographic-related differences in sleep attitudes existed and whether demographic differences in sleep attitudes predicted sleep outcomes. This information helped examine whether sleep attitudes are more relevant for some groups than for others. If sleep attitudes are potentially modifiable health behaviors, it will be important to know whether the importance placed on assessing and addressing sleep attitudes in future sleep interventions should be differentially emphasized for different demographic groups.

The authors recognize the need for caution when interpreting these data since this study was mostly exploratory in nature. No causal inferences can be made about the data since the present study was cross-sectional. Future research should work to build on this study by potentially experimentally modifying sleep attitudes or examining sleep attitudes before and after an intervention aimed at changing them.

## Implications and conclusions

Overall, it appears that positive sleep attitudes were associated with better sleep, although not uniformly across all groups. It is important to discern for whom positive sleep attitudes are beneficial, and whether other demographic factors interact with sleep attitudes, modifying its relative importance for sleep outcomes. This is especially crucial given that some demographic groups experience disparities in health (e.g. Williams et al., 2010) and sleep outcomes (e.g. Williams et al., 2016). A logical next step in the study of sleep attitudes is to explore whether and under what circumstances they can be altered, and whether improving sleep attitudes results in short- or long-term improvement in sleep outcomes. Previous research regarding health attitudes and behavior has demonstrated that positive attitudes can lead to favorable outcomes, such as attitudes and behaviors related to skin cancer and sun exposure (Mermelstein & Riesenberg, 1992), and that brief interventions to alter attitudes toward personal lifestyle habits have been successful (Shahar et al., 2009). Therefore, it may be possible to explore specific interventions aimed at altering and improving sleep attitudes and examining changes in sleep outcomes.

Preliminary evidence suggests that attitudes may be a better predictor of sleep outcomes than sleep knowledge (Peach et al., 2018). If this finding is supported by future studies, researchers and clinicians should consider assessing sleep attitudes, and address how such attitudes may underlie sleep practices. Although the present data suggest that race/SES differences in attitudes may exist, additional information is needed on the association between attitudes and sleep outcomes. Positive sleep attitudes may not be consistently beneficial, or may vary as a function of which sleep practice or outcome is targeted. Research that contributes to understanding the role of sleep attitudes as the underpinning of sleep outcomes could have important implications for using sleep attitudes as a potentially adaptable prevention strategy for sleep and better overall health.

## Appendix

Table A1. Three way interactions on sleep hygiene.

**Table:**

Model	Outcome variable	*b*	S.E.	*R*^2^	Δ*R*^2^
Step 1				.36^b^	.36^b^
	(Intercept)	82.17	3.16		
	Age	.12	.17		
	Gender	1.84	3.24		
	SES	–.18	.88		
	Race	–6.70^a^	3.38		
	Sleep Attitudes	–15.00^b^	1.71		
Step 2				.39^b^	.03
	(Intercept)	80.53	3.19		
	Age	.08	.16		
	Gender	1.07	3.20		
	SES	–.63	.91		
	Race	–4.92	3.43		
	Sleep Attitudes	–18.80^b^	3.17		
	Gender X Sleep Attitudes	7.03^b^	3.35		
	SES X Sleep Attitudes	–1.61^c^	.88		
	Race X Sleep Attitudes	1.88	3.52		
	Age X Sleep Attitudes	.23	.21		
	Gender X Race	–3.31	7.19		
	Gender X SES	–.05	2.07		
	Gender X Age	–.22	.35		
	SES X Race	–1.29	2.02		
	Age X Race	–.33	.50		
	Age X SES	.05	.10		
Step 3				.40^b^	.01
	(Intercept)				
	Age	.29	.81		
	Gender	2.85	7.72		
	SES	.70	3.14		
	Race	–3.76	5.72		
	Sleep Attitudes	–19.68^b^	5.16		
	Gender X Sleep Attitudes	9.20	7.20		
	SES X Sleep Attitudes	–1.16	–1.16		
	Race X Sleep Attitudes	3.73	5.78		
	Age X Sleep Attitudes	.07	.71		
	Gender X Race	–2.98	8.80		
	Gender X SES	–2.30	4.41		
	Gender X Age	.05	1.21		
	SES X Race	–2.15	3.41		
	Age X Race	–.15	.87		
	Age X SES	–.05	.31		
	Gender X Race X SES	3.34	5.14		
	Gender X Race X Sleep Attitudes	–3.35	8.41		
	Gender X SES X Sleep Attitudes	–.52	2.32		
	Race X SES X Sleep Attitudes	–.13	2.41		
	Age X Race X Sleep Attitudes	.03	.69		
	Age X Race X SES	.10	.34		
	Age X Race X Gender	–.33	1.29		
	Age X SES X Gender	.22	.46		
	Age X SES X Sleep Attitudes	.02	.14		

Notes: *N* = 172. ^a^*p* < .05; ^b^*p* < .01; ^c^*p* = .06. *b* = unstandardized regression weight; Δ*R*^2 ^= Change in *R*^2^ from prior model.

Table A2. Three way interactions on sleep duration (weeknights).

**Table:**

Model	Outcome variable	*b*	S.E.	*R*^2^	Δ*R*^2^
Step 1				.12^b^	.12^b^
	(Intercept)	.74	.10		
	Age	.15^b^	.01		
	Gender	.15	.11		
	SES	–.03	.03		
	Race	–.23^a^	.11		
	Sleep Attitudes	–.20^a^	.06		
Step 2				.14^b^	.02
	(Intercept)	.66	.13		
	Age	–.01	.02		
	Gender	.36^c^	.20		
	SES	.02	.06		
	Race	–.13	.16		
	Sleep Attitudes	–.23^c^	.12		
	Gender X Sleep Attitudes	–.02	.12		
	SES X Sleep Attitudes	.04	.03		
	Race X Sleep Attitudes	.06	.13		
	Age X Sleep Attitudes	–.01	.01		
	Gender X Race	–.41	.24		
	Gender X SES	–.01	.07		
	Gender X Age	–.01	.01		
	SES X Race	–.05	.07		
	Age X Race	.03	.02		
	Age X SES	–.01^b^	.01		
Step 3				.20^b^	.06
	(Intercept)	.79	.16		
	Age	.02	.03		
	Gender	.27	.25		
	SES	–.12^a^	.10		
	Race	–.21	.18		
	Sleep Attitudes	–.33^a^	.16		
	Gender X Sleep Attitudes	.20	.23		
	SES X Sleep Attitudes	.09	.07		
	Race X Sleep Attitudes	.01	.18		
	Age X Sleep Attitudes	–.02	.02		
	Gender X Race	–.31	.28		
	Gender X SES	.20	.14		
	Gender X Age	–.02	.04		
	SES X Race	.11	.11		
	Age X Race	.01	.03		
	Age X SES	–.02	.01		
	Gender X Race X SES	–.30	.16		
	Gender X Race X Sleep Attitudes	–.11	.27		
	Gender X SES X Sleep Attitudes	–.06	.07		
	Race X SES X Sleep Attitudes	.00	.07		
	Age X Race X Sleep Attitudes	–.01	.02		
	Age X Race X SES	.02	.01		
	Age X Race X Gender	.02	.04		
	Age X SES X Gender	.01	.01		
	Age X SES X Sleep Attitudes	.01	.01		

Notes: *N* = 172. ^a^*p* < .05; ^b^*p* < .01. *b* = unstandardized regression weight; Δ*R*^2 ^= Change in *R*^2^ from prior model.

Table A3. Three way interactions on sleep duration (weekends).

**Table:**

Model	Outcome variable	*b*	S.E.	*R*^2^	Δ*R*^2^
Step 1				.15^b^	.15^b^
	(Intercept)	82.17	3.16		
	Age	.01^b^	.01		
	Gender	1.84	3.24		
	SES	–.18	.88		
	Race	–6.70^a^	3.38		
	Sleep Attitudes	–15.00^b^	1.71		
Step 2				.20^b^	.05^a^
	(Intercept)	80.53	3.19		
	Age	.08^b^	.16		
	Gender	1.07	3.20		
	SES	–0.63	.91		
	Race	–4.92	3.43		
	Sleep Attitudes	–18.80^b^	3.17		
	Gender X Sleep Attitudes	7.03^b^	3.35		
	SES X Sleep Attitudes	–1.61^c^	.88		
	Race X Sleep Attitudes	1.88	3.52		
	Age X Sleep Attitudes	–.01	.01		
	Gender X Race	–.45	.24		
	Gender X SES	.03	.07		
	Gender X Age	–.01	.01		
	SES X Race	.01	.07		
	Age X Race	.03	.02		
	Age X SES	–.01^b^	.01		
Step 3				.41^b^	.21^b^
	(Intercept)	.55	.15		
	Age	.04	.02		
	Gender	.25	.23		
	SES	–.19^a^	.09		
	Race	–.17	.17		
	Sleep Attitudes	–.38^a^	.15		
	Gender X Sleep Attitudes	–.13	.21		
	SES X Sleep Attitudes	.09	.07		
	Race X Sleep Attitudes	–.02	.17		
	Age X Sleep Attitudes	.00	.02		
	Gender X Race	–.17	.26		
	Gender X SES	.24	.13		
	Gender X Age	–.06	.04		
	SES X Race	.18	.10		
	Age X Race	–.02	.03		
	Age X SES	–.01a	.01		
	Gender X Race X SES	–.41	.15		
	Gender X Race X Sleep Attitudes	.52^a^	.25		
	Gender X SES X Sleep Attitudes	–.13^a^	.07		
	Race X SES X Sleep Attitudes	.01	.07		
	Age X Race X Sleep Attitudes	–.03	.02		
	Age X Race X SES	.01	.01		
	Age X Race X Gender	.06	.04		
	Age X SES X Gender	.01	.01		
	Age X SES X Sleep Attitudes	.01	.01		

Notes: *N* = 172. ^a^*p* < .05; ^b^*p* < .01. *b* = unstandardized regression weight; Δ*R*^2 ^= Change in *R*^2^ from prior model.

Table A4. Three way interactions on sleep quality (weeknights).

**Table:**

Model	Outcome variable	*b*	S.E.	*R*^2^	Δ*R*^2^
Step 1				.15^b^	.15^b^
	(Intercept)	5.75	.57		
	Age	.03	.03		
	Gender	1.31^a^	.59		
	SES	–.03	.16		
	Race	–.87	.61		
	Sleep Attitudes	–1.44^a^	.31		
Step 2				.20^b^	.05
	(Intercept)	5.24	.76		
	Age	–.07	.09		
	Gender	1.61	1.07		
	SES	.35	.35		
	Race	–.22	.88		
	Sleep Attitudes	–1.89^b^	.64		
	Gender X Sleep Attitudes	.96	.66		
	SES X Sleep Attitudes	.14	.18		
	Race X Sleep Attitudes	.01	.68		
	Age X Sleep Attitudes	–.03	.04		
	Gender X Race	–.68	1.32		
	Gender X SES	–.27	.38		
	Gender X Age	–.09	.06		
	SES X Race	–.39	.37		
	Age X Race	.17	.09		
	Age X SES	–.01	.02		
Step 3				.22^b^	.02
	(Intercept)	5.33	.92		
	Age	–.05	.15		
	Gender	1.79	1.39		
	SES	–.11	.57		
	Race	–.22	1.04		
	Sleep Attitudes	–2.42^a^	.94		
	Gender X Sleep Attitudes	2.15	1.31		
	SES X Sleep Attitudes	.09	.42		
	Race X Sleep Attitudes	.52	1.05		
	Age X Sleep Attitudes	–.11	.13		
	Gender X Race	–1.09	1.59		
	Gender X SES	.29	.80		
	Gender X Age	–.13	.22		
	SES X Race	.04	.62		
	Age X Race	.15	.16		
	Age X SES	–.07	.06		
	Gender X Race X SES	–.78	.93		
	Gender X Race X Sleep Attitudes	–1.24	1.53		
	Gender X SES X Sleep Attitudes	.47	.42		
	Race X SES X Sleep Attitudes	–.10	.44		
	Age X Race X Sleep Attitudes	.06	.12		
	Age X Race X SES	.06	.06		
	Age X Race X Gender	.04	.23		
	Age X SES X Gender	.05	.08		
	Age X SES X Sleep Attitudes	.02	.03		

Notes: *N* = 172. ^a^*p* < .05; ^b^*p* < .01. *b* = unstandardized regression weight; Δ*R*^2 ^= Change in *R*^2^ from prior model.

Table A5. Three way interactions on sleep quality (weekends).

**Table:**

Model	Outcome variable	*b*	S.E.	*R*^2^	Δ*R*^2^
Step 1				.17^b^	.15^b^
	(Intercept)	5.44	.55		
	Age	.02	.03		
	Gender	1.45^a^	.57		
	SES	–.05	.15		
	Race	–.78	.59		
	Sleep Attitudes	–1.52^a^	.73		
Step 2				.23^b^	.06
	(Intercept)	4.79	.73		
	Age	–.06	.08		
	Gender	1.92	1.03		
	SES	.24	.34		
	Race	.03	.85		
	Sleep Attitudes	–2.39^b^	.61		
	Gender X Sleep Attitudes	1.05	.63		
	SES X Sleep Attitudes	.12	.18		
	Race X Sleep Attitudes	.62	.65		
	Age X Sleep Attitudes	–.02	.04		
	Gender X Race	–.94	1.26		
	Gender X SES	–.16	.36		
	Gender X Age	–.10	.06		
	SES X Race	–.37	.36		
	Age X Race	.16	.09		
	Age X SES	–.02	.02		
Step 3				.26^b^	.03
	(Intercept)	5.05	.88		
	Age	–.02	.14		
	Gender	1.95	1.33		
	SES	–.19	.54		
	Race	–.14	.99		
	Sleep Attitudes	–2.64^a^	.89		
	Gender X Sleep Attitudes	1.59	1.24		
	SES X Sleep Attitudes	.18	.40		
	Race X Sleep Attitudes	.65	.99		
	Age X Sleep Attitudes	–.09	.12		
	Gender X Race	–1.24	1.52		
	Gender X SES	.43	.76		
	Gender X Age	–.15	.21		
	SES X Race	.05	.59		
	Age X Race	.12	.15		
	Age X SES	–.06	.05		
	Gender X Race X SES	–.98	.89		
	Gender X Race X Sleep Attitudes	–.10	1.46		
	Gender X SES X Sleep Attitudes	.42	.40		
	Race X SES X Sleep Attitudes	–.20	.42		
	Age X Race X Sleep Attitudes	.04	.12		
	Age X Race X SES	.05	.06		
	Age X Race X Gender	.05	.22		
	Age X SES X Gender	.04	.08		
	Age X SES X Sleep Attitudes	.03	.02		

Notes: *N* = 172. ^a^*p* < .05; ^b^*p* < .01. *b* = unstandardized regression weight; Δ*R*^2 ^= Change in *R*^2^ from prior model.
